# Control methods for invasive mosquitoes of *Aedes aegypti* and *Aedes albopictus* (Diptera: Culicidae) in Indonesia

**DOI:** 10.14202/vetworld.2023.1952-1963

**Published:** 2023-09-23

**Authors:** Muhammad Rasyid Ridha, Lenie Marlinae, Tien Zubaidah, Noor Ahda Fadillah, Junus Widjaja, Dian Rosadi, Nita Rahayu, Murtiana Ningsih, Iwan Desimal, Arif Sofyandi

**Affiliations:** 1 Vector-borne and Zoonotic Diseases Research Group, Research Center for Public Health and Nutrition, Cibinong Science Center, National Research and Innovation Agency, Jl. Raya Jakarta-Bogor KM.46, Bogor, West Java, 16915, Indonesia; 2 Department of Environmental Health, Public Health Study Program, Medical Faculty, Universitas Labung Mangkurat, Jl. A. Yani, Km. 36 Banjarbaru, South Kalimantan, Indonesia; 3 Environmental Health Program, Banjarmasin Health Polytechnic, Jl. H. Mistar Cokrokusumo No.1A, Kemuning, Banjar Baru, South Kalimantan, 70714, Indonesia; 4 Department of Epidemiology, Public Health Study Program, Medical Faculty, Universitas Labung Mangkurat, Jl. A. Yani, Km. 36 Banjarbaru, South Kalimantan, Indonesia; 5 Public Health Study Program, Sports Sciences and Public Health Faculty, Universitas Pendidikan Mataram, Jl. Pemuda No. 59 A Mataram West Nusa Tenggara, Indonesia

**Keywords:** *Bacillus thuringiensis*, chikungunya viruses, dengue, Indonesia

## Abstract

The two invasive mosquito species in Indonesia are *Aedes aegypti* and *Ae. albopictus*. These mosquitoes are a serious nuisance to humans and are also the primary vectors of several foreign pathogens, such as dengue, Zika, and chikungunya viruses. Efforts must be made to reduce the possibility of mosquito bites and the potential for disease transmission. Given the invasion of these two *Aedes* species, this approach should be considered as part of an integrated strategy to manage them. This review discusses existing and developing control techniques for invasive *Ae. aegypti* and *Ae. albopictus*, with an emphasis on those that have been and are being used in Indonesia. Environmental, mechanical, biological (e.g., *Bacillus thuringiensis* and *Wolbachia*), and chemical (e.g., insect growth regulators and pyrethroids) approaches are discussed in this review, considering their effectiveness, sustainability, and control methods.

## Introduction

Increased worldwide trade and distribution, driven by human migration and environmental changes, promote the introduction and establishment of invasive mosquito species (IMS) beyond their geographic range. Mosquitoes of the *Ae. aegypti* genus (Diptera: Culicidae) can be highly invasive because their eggs can survive for months in desiccated conditions and withstand long-term transport [1]. Two species have been defined in Indonesia, namely, *Ae. albopictus* and *Ae. aegypti*.

The impact of these mosquitoes on human health is partly due to their rapid and aggressive spread from their native range on islands in East Asia, the West Pacific, and the Indian Ocean. *Ae. albopictus* has been identified on every continent except Antarctica for the past 30–40 years [2]. It was first discovered in Europe (Albania) in 1979 and has since spread to almost all European countries, extending into Turkey and the Middle East before moving northward. *Ae. albopictus* is most prevalent in Italy and southern France but has limited distribution in the Netherlands, southern Switzerland, Germany, Bulgaria, Belgium, and Russia. These observations confirm the distribution predictions related to climate change [3, 4]. There is significant evidence showing that *Ae. aegypti* originated in Africa, with several sub-Saharan African forests, basins, and other natural water bodies serving as breeding grounds [5]. Evidence from DNA sequencing and large-scale single-nucleotide polymorphism analysis suggests that this species may have spread across the western Pacific to Asia and Australia following its introduction to the New World [6]. This implies that New World populations are directly descended from African and Asian/Australian populations [4].

Invasive mosquito species are characterized by their propensity to invade new regions, negatively impact human and animal health, and adversely affect the environment and local economy. They are a significant nuisance to humans due to their aggressive biting behavior, which interferes with social interactions and outdoor activities [7]. Moreover, they are efficient at spreading various foreign diseases (e.g., dengue and chikungunya viruses) and increasing the risk of epidemics in Indonesia by establishing and introducing these pathogens into the country through infections carried by travelers [8].

Dengue virus infection was first reported in Indonesia in 1968 in Jakarta and Surabaya (East Java), followed by Bandung (West Java) and Yogyakarta. Since then, suspected cases of dengue have been recorded by the Ministry of Health. In the early 1980s, the annual case count increased from 10,000 to 30,000, and over the last decade, the reported incidence has ranged from 30,000 to 60,000 cases/year [9]. Notably, significant peaks were reported in 1973 and 1988. At present, dengue has spread to all 35 provinces in Indonesia, with annual case numbers ranging from 10,000 to 25,000 [10]. Conversely, chikungunya was first discovered in Indonesia in the 1970s. Initial outbreaks occurred in South Sumatra, Java, and West Kalimantan. From these regions, the virus spread to Sulawesi, Nusa Tenggara, and Papua. This plague usually strikes every 20 years [11].

**Figure-1:**
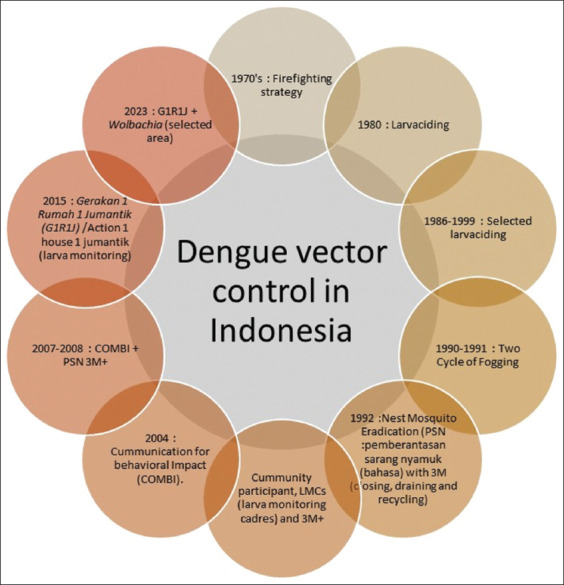
Dengue vector control in Indonesia from 1970 to 2023 (modification from Sulistyawati [15]).

The prevalence of these diseases is closely related to the spread of these mosquitoes in Indonesia. Control measures have been implemented using insecticides and community empowerment initiatives. These measures have evolved over time, including larvicidation in the 1980s, followed by selective larvicidation from 1986 to 1989, two fogging cycles, distribution of 3M mosquito nets (covering, burying, and draining), introduction of 3M Jumantik, and the implementation of Communication Behavior Impact (COMBI) in 2004 [12]. Subsequently, strategies such as Pemberantasan sarang nyamuk or “Eradication of mosquito nests” + COMBI were employed, and from 2015 onward, the G1R1J (1 House 1 Jumantik Movement) initiative has been in place. Implementation of Wolbachia technology in certain locus areas has begun in 2023, namely in Semarang, West Jakarta, Bandung, Kupang, and Bontang (Figure-1) [13–15].

In this review, we discuss existing and developing control methods for IMS, with a focus on their application in Indonesia. We categorize these control measures into four categories based on the World Health Organization (WHO) guidelines: Environmental, mechanical, biological, and chemical. We describe the effectiveness, ecological impact, sustainability, and development stages of each management approach, aiming to limit the impact and spread of *Ae. aegypti* and *Ae. albopictus*. Personal protection measures, such as insect repellents, the use of protective clothing, or mosquito nets are not included in this review despite their effectiveness in preventing mosquito bites and implementation in the community. However, these methods effectively protect individuals from pathogen transmission.

This review discusses existing or developing control techniques for the invasive mosquitoes *Ae. aegypti* and *Ae. albopictus*, focusing on control measures already in use in Indonesia. Environmental, mechanical, biological (e.g., *Bacillus thuringiensis* (*Bti*) and *Wolbachia*), and chemical (insect growth regulators and pyrethroids) approaches are discussed when considering effective methods of efficiency, sustainability, and control.

## Environmental Methods

One of the key strategies for controlling IMS is to reduce habitat resources that can serve as potential breeding sites, such as various containers, ranging from bottle caps to water tanks. Even hidden places, such as water reservoirs in refrigerators and dispensers, need to be considered. The aim is to either relocate or seal off temporary water reservoirs, bury them in the ground if not in use, or recycle them into reusable materials [16]. This method is often the initial approach for controlling mosquitoes that breed in artificial containers, such as the *Aedes* species. Resource restriction campaigns generally accomplish temporary suppression of *Aedes* mosquitoes by reducing available oviposition sites. Moreover, this approach can alter the distribution of native mosquitoes, such as *Culex* spp., in a given area.

Invasive mosquito species can breed in various urban, suburban, and rural habitats. *Aedes albopictus* favors natural containers, such as bamboo and coconut fruit, whereas *Ae. aegypti* prefers artificial containers [17, 18]. Research conducted in Bogor City (West Java, Indonesia) revealed that 74.4% of *Ae. albopictus, Ae. aegypti, Aedes* spp., *Armigeres subalbatus*, and *Culex quinquefasciatus* were found on bromeliads of the genera *Neoregelia, Cryptanthus*, and *Alcantarea* [19]. Other studies have identified that *Ae. albopictus* prefers disposable containers by 54.77%, such as cans, used bottles, buckets, fish ponds, bamboo stumps, tires, and drums [19]. The current implementation of 3M Plus under the Health Authority program in Indonesia includes sealing off water reservoirs, draining water infected with larvae, and burying used items to prevent them from becoming larvae habitats. The “Plus” aspect involves additional activities such as sprinkling larvicidal powder on water reservoirs that are difficult to clean, using insect or mosquito repellent, and utilizing mosquito nets while sleeping. Research has shown that 3M Plus efforts have influenced the rate of dengue incidents [20].

Since certain breeding sites can be extremely productive for specific mosquito species, the type of housing and habitat accessible in a given area is closely linked to mosquito production. For instance, abandoned boats near Kurri Caddi’s shore are among *Ae. aegypti*’s most productive breeding grounds. Receptacle clusters also serve as “hot spots” for mosquito breeding and act as sources of infestation within neighborhoods. Consequently, concentrating on the most productive breeding areas is the most efficient use of time and resources. A metal drum known as the “Anti-dengue Hat” and a nylon net were used in Tarakan, northern Kalimantan, as part of a resource reduction campaign against *Ae. aegypti*. These items were previously recognized as the most productive breeding grounds.

The type of habitat present in a region directly affects mosquito production because certain breeding sites can be extremely productive for specific mosquito species. For instance, as one of the most productive breeding sites, *Ae. aegypti* thrives in used boats in Kurri Caddi (South Sulawesi) [21]. In addition, clusters of containers, which serve as “hot spots,” influence mosquito production and act as a source of infestation in the surrounding vicinity. Therefore, focusing reduction efforts on the most productive breeding sites is the most efficient use of time and money. A resource reduction campaign against *Ae. aegypti* was conducted in Tarakan, northern Kalimantan, which used a nylon net to cover water tanks and a metal drum known as the “*Topi Anti-Dengue*” or “Anti-dengue Hat.” These items had previously been identified as the most fruitful breeding sites in the study area [22].

Effective resource reduction, particularly for *Ae. aegypti* and *Ae. albopictus*, requires actions such as brushing to remove deposited eggs or meticulous and frequent maintenance of containers in daily use. Achieving this requires collaboration with homeowners [23]. Public awareness campaigns and health promotion efforts aimed at assisting communities in identifying and removing small water containers from their properties have become essential components of mosquito control programs. This is because private residences are a significant source of *Aedes* mosquito habitats. However, these efforts are often insufficient to persuade residents to reduce these habitats and often require regulation and assistance from local government authorities. A sustainable, community-based approach that can significantly reduce the cost of control measures involves enhancing resource reduction by focusing on containers in and around households [23].

## Mechanical Methods

### BG-Sentinel (BGS) traps with odor bait

Surveys and population monitoring are methods employed to capture mosquito eggs and adult mosquitoes. To control adult mosquito populations, studies have suggested using mass traps with odor baits [24]. The available techniques for trapping *Aedes* mosquitoes target host-seeking female mosquitoes (e.g., BGS traps) or gravid females (e.g., ovitraps or sticky/gravid traps; Biogents AG, Regensburg, Germany).

An ovitrap is a tool that detects the presence of *Ae. aegypti* and *Ae. albopictus* mosquitoes when the population density is low. This device is commonly used to detect the onset of a new infestation and monitor the density of dengue vector populations following containment efforts. The ovitrap capitalizes on the *Aedes* mosquitoes’ tendency to lay eggs in small containers. It is a cost-effective, sensitive, and passive monitoring device used to locate mosquito breeding sites in containers and gauge the dynamics of adult populations. The incorporation of a fatal mechanism in ovitraps allows for their long-term use, with minimal risk of becoming sources of adult mosquito production [25]. Egg-laying strips laced with pesticides (e.g., permethrin and deltamethrin) were used to test lethal ovitraps. Although not statistically supported, field tests conducted in Semarang and Salatiga (Indonesia) using lethal ovitraps revealed a decrease in *Ae. aegypti* population density [26, 27]. According to a field study, lethal ovitrap control programs significantly impacted *Ae. aegypti* populations in Australia and were well received by the public [28]. In addition, organic infusions, such as grass, straw, and nitrogen–phosphorus–potassium fertilizers, can be used to make ovitraps more attractive [29]. Combining these traps with oviposition stimulants may further enhance mosquito population management. Sticky ovitraps and gravid traps with surface adhesive have also been developed to survey gravid females, with several designs field-tested to decrease *Aedes* mosquito populations [30].

The use of BGS mosquito traps for dengue monitoring is still limited in Indonesia. The BGS trap replicates the convection currents created by the human body, employs enticing visual cues, and releases artificial skin emanations through a large surface area. While it can operate without CO_2_, it is particularly effective when used with CO_2_ to capture mosquitoes. In Yogyakarta, BGS has been used to evaluate the population density of *Ae. aegypti* and *Ae. albopictus* in trial areas and study the implications for *Wolbachia*. The study identified areas with a consistently high mosquito population and factors affecting the decline in mosquito populations, including vegetation. These results aided in formulating strategies for introducing *Wolbachia* or similar approaches aimed at suppressing mosquito populations in all tested locations [31].

Several studies have shown that adding an attractant, such as CO_2_, to BGS traps increases their effectiveness in capturing *Aedes* mosquitoes. The use of BGS traps has been tested as an *Ae. albopictus* management strategy in northern Italy. In areas where traps were deployed, ranging from one trap per 150 m^2^ to 350 m^2^, human bite rates were lower than control locations [32]. Thus, BGS traps were considered a promising tool that could be employed in STI control programs or as a driving component of a push–pull strategy, despite their use being constrained by their electrical power requirements. The push–pull strategy, which combines a repellent with an attractive stimulant, has been successful in managing various agricultural pests and is now being proposed as a mosquito control method [33].

During outbreaks, countermeasures are put into place through larvicidation with temefos and mass fumigation using chemical insecticides. More recently, *Bti* liquid has been used, although it is comparatively more expensive. The Aedes mosquito population in Indonesia is becoming increasingly resistant to chemical insecticides [34]. In Makassar, Indonesia, implementing a fogging program before the transmission period has significantly reduced the *Aedes* population compared to waiting for cases to occur [35].

## Chemical Methods

Pyrethroids as chemical adulticides: Space spraying, indoor residual spraying (IRS), insecticide-treated surfaces, and attractive lethal sugar baits.

### Space spraying

Space spraying involves dispersal of a liquid fog of insecticide outdoors to kill adult insects. It is a crucial method for controlling *Aedes* mosquitoes and mitigating arboviral diseases [36], especially during dengue fever epidemics. Space spraying aims to incapacitate and kill adult insects by spreading a mist of insecticide droplets in an area [37].

Rebellion using fogging is considered the most appropriate method by society. However, fogging is employed only when necessary, as many negative events have occurred due to its toxicity. Thus, this approach is not always the best course of action, as its primary purpose is to kill infectious adult mosquitoes carrying the dengue virus [38]. While this method can effectively reduce mosquito populations below elimination thresholds in regions with low-to-moderate transmission rates, additional control measures are needed in areas with high transmission rates or more resilient vector species. Space spraying can play a crucial role in reducing transmission under such conditions by influencing the behavior of outdoor mosquitoes, which are more resistant to insecticide-treated nets (ITNs) and IRS. This is especially important in the current and future climate as the range of ITNs increases and the dispersal of exophagic and zoophilic vector species gains importance [37]. The insecticides used for ITNs in Indonesia belong to the pyrethroid group, namely, permethrin and deltamethrin [39, 40].

The implementation of space spraying in Indonesia started in response to the reports of dengue fever cases. When cases of dengue were reported, they were forwarded to the Health Service, which collaborated with the Health Department to gather information on these cases and subsequently conducted epidemiological investigations. The main objective of these investigations was to determine whether any additional dengue cases existed and to assess the potential for widespread disease transmission in the region. The results of these epidemiological investigations will then inform the next steps to eradicate dengue. When a positive case of dengue fever was identified, the Health Service initiated fogging measures, accompanied by the dissemination and harassment of mosquitoes. Typically, fogging operations are initiated when there is evidence of dengue transmission in a specific area. Fogging is conducted within a radius of at least 200 m and in two cycles with a 1-week interval. In addition, these fogging activities are performed in coordination with local public health centers [41].

### Indoor residual spraying

Indoor residual spraying is an important strategy for mosquito prevention and control. In IRS, pyrethroid pesticides are primarily used for controlling *Ae. aegypti* in emergencies. Indoor residual spraying was found to be more effective than outdoor spraying in small-scale studies for reducing *Ae. aegypti* populations. This is because adult *Ae. aegypti* mosquitoes often reside indoors, where food, mates, and oviposition substrates are easily available [42]. These strategies have proven highly effective in reducing the disease burden in various epidemiological contexts [43].

This process involves spraying the walls of houses with an insecticide that remains on the sprayed surfaces. The residual potential was assessed through the WHO-recommended bioassay test [44]. Bioassay testing helps determine the effectiveness of insecticides used in vector control programs. It aims to measure the killing power of the insecticide and to analyze the effects of the residues, as well as assess the quality of the insecticide used [45]

In Indonesia, IRS is initiated on receiving reports of dengue cases in some regions. Indoor residual spraying using ultralow volume is conducted within a radius of at least 200 m and implemented in two cycles with a 1-week interval. This activity is coordinated with local public health centers (Puskesmas) [41].

### Insecticide-treated materials

The use of insecticide-treated materials shows promise in reducing recent dengue vector infestations at the household level [41]. One such material is long-lasting insecticidal nets (LLINs), which can be used as curtains on doors and windows. Long-lasting insecticidal nets significantly impact *Ae. aegypti* when used near humans and can remain entomologically significant for up to 2 years post-insertion, even in *Aedes* populations that exhibit resistance to pyrethroids [46]. These mosquito nets form a protective barrier around individuals sleeping under them. Notably, ITNs provide greater protection than non-insecticide-treated mosquito nets [42].

Mosquitoes and other insects can die on exposure to the insecticides used in LLINs. In addition, insecticides can repel mosquitoes, reducing the number of mosquitoes that enter the house. Achieving high community coverage with LLINs can decrease the mosquito population and their lifespan, as well as protect all community members, whether they use mosquito nets or not. However, to achieve these results, more than 50% of the population in the community must use ITNs [42].

At present, only two classes of insecticides (pyrrole and pyrethroid) are permitted for use in ITNs due to their minimal health risks to humans. These insecticides, which are generally toxic to insects, are highly effective but may lose their effectiveness in 6–12 months if the nets are washed frequently. Re-treatment involves simply dipping the nets in a water and insecticide solution and drying them in the shade. In endemic countries, the need for frequent re-treatment is a significant barrier to the widespread use of ITNs [42].

In Indonesia, the use of fitting LLINs is an effective approach for preventing *Aedes* mosquito bites, especially in pregnant mothers, babies, and infants. Fitting LLINs serve as a physical barrier against mosquitoes, and their insecticide effectively kills mosquitoes. Insect-destroyed cloves are typically given free of charge to communities in dengue-endemic areas [47].

### Attractive toxic sugar bait (ATSB)

One method developed for controlling *Aedes* mosquitoes is the ATSB strategy, which employs an attractant (e.g., flower accents and/or fruit juices), a phagostimulant (sugar), and an oral insecticide like boric acid (H_3_BO_3_), which is a low-toxicity, chemically stable, and inorganic insecticide [48]. This promising “attract-and-kill” strategy for mosquito control employs flower nectar or fruit juice to attract mosquitoes, a sugar solution to stimulate feeding, and poison to kill mosquitoes [49].

The ATSB strategy, attractive, and lethal reduces reliance on chemical pesticides and is based on mosquitoes deriving their energy from plant sugars found in floral sources (e.g., nectar and fruit juice). Mosquitoes locate these sources through a combination of visual and olfactory cues. Certain olfactory receptors respond to specific odors and require coreceptors for odor recognition. In addition, ionotropic receptors distinguish various classes of chemical compounds, including amines, aldehydes, ketones, and carboxylic acids [49].

In Indonesia, ATSB generally uses red sugar as an attractant. One attraction method involves a solution of red sugar and yeast fermentation. Sugar is a commonly used ingredient in the fermentation process. The reaction of red sugar and yeast produces CO_2_, which is one of the attractants that appeal to the sensory receptors of *Ae. aegypti* mosquitoes. Consequently, the sugar fermentation can lure mosquitoes closer to the bait trap [43].

## Biological Methods

### Entomopathogenic fungi

Biological control using entomopathogenic fungi, such as *Beauveria bassiana* and *Metarhizium anisopliae*, presents a viable method for managing adult and pre-adult mosquito populations. In laboratory experiments, *B. bassian*a demonstrated the ability to reduce the lifespan of *Ae. aegypti*. Semi-field trials have further shown its effectiveness in reducing fecundity, adult survival, and blood-feeding ability in *Ae. aegypti* [50]. Scientific evidence supports the biolarvicidal and adulticidal activities of *M. anisopliae* against *Aedes* mosquitoes [51]. Soil isolates of *M. anisopliae* from Sumatra, Indonesia, are known to be pathogenic to the eggs, larvae, and adults of *Ae. aegypti*. Notably, the highest egg mortality was caused by the *M. anisopliae* MSwTp3 isolate (38.31%). A new finding from this study is that exposure to the fungus kills eggs and can continue to eliminate newly emerged larvae, pupae, and adults [51]. In addition, modifications were made with a mixed formulation of *M. anisopliae* and olive oil, capable of achieving a 100% mortality rate for *Ae. aegypti* when applied to ovitraps [52]. *Beauveria bassiana* isolates were tested to determine the lethal contamination (LC) 50 concentration. This resulted in *Ae. aegypti* mortality, with 49 × 10^9^ spores/mL and 19.0 × 10^8^ spores/mL recorded at 24 h and 48 h, respectively. Furthermore, the LC50 values at 24 h and 48 h for *Ae. aegypti* were 1.07–107 spores/mL and 1.49 –105 spores/mL, respectively [53]. In addition, *B. bassiana* has proven effective against *Ae. albopictus* in the third instar larvae, where a larvicidal formula of the enzyme compound *B. bassiana* and chitinase was effectively used as a larvicide [54]. This novel approach could serve as a promising basis for practical and economical strategies to reduce viable *Aedes* mosquito egg populations.

### *Bacillus thuringiensis* var. israelensis as microbial larvicides

Dichlorodiphenyltrichloroethane has played a crucial role in controlling many vector-borne infectious diseases for about 40 years. However, its use can damage the environment due to its persistent nature [55]. At present, eco-friendly poison-based bioinsecticides, namely, *Bti* and *B. sphaericus* (*Bs*), are employed to selectively target Culicidae larvae, such as mosquitoes, without harming other flora and fauna.

The World Health Organization recommends Larva Source Management (LSM) as an additional approach in integrated vector control, complementing the use of insecticides and community empowerment initiatives [56]. Unlike most chemical larvae repellents, *Bti* exhibits low resistance potential, and no loss of efficacy has been observed following field applications. Although *Bti* resistance has been reported, it appears to be less significant in field applications, especially when *Bs* is combined with *Bti* [57]. Depending on environmental conditions, especially the number, size, and accessibility of breeding sites, *Bti*-based larvicides can significantly aid population management in integrated control programs. Larvae are found near their breeding sites and are easily accessible in many environments, enabling LSM to significantly reduce adult mosquito populations [58–60].

However, these larvicides have certain limitations in tropical Africa and require careful field testing before being employed in vector control. Elevated temperatures increase larvicide effectiveness, partly due to increased larval feeding rates [61]. However, they can also increase biodegradation [62]. Factors such as dilution from heavy rainfall, wind flow over water surfaces, and interaction with flora and fauna can impact larvicide efficiency and residual activity. In addition, thermal conditions can drastically shorten the reproductive cycle of mosquito vectors, considerably affecting the need to re-treat breeding sites.

Each geographic area presents a unique combination of these factors and emphasizes the need to test larvicide efficacy under their respective field conditions. This is the first attempt at *Bti* testing in the region as part of a large-scale intervention program using satellite risk maps to monitor larval densities [63]. *Bacillus thuringiensis* serovar israelensis is a natural bioinsecticide that effectively controls mosquito larvae by Flacio *et al*. [64] producing a toxin that disrupts mosquito larval digestion [65]. Its long-term use is safe and does not induce resistance [66]. The initial stage involved a laboratory experiment with five doses in the treatment and control groups. The test results showed that the *Bti* H-14 was effective in killing *Ae. aegypti* at doses of 50 μL and 40 μL/2.5 L of water [67]. A study in India yielded similar results, with effective *Bti* use in purified water at a dose of 1 mL/50 μL resulting in mortality within 10–17 days [68]. Another study identified an effective dose of 8 mg/L for Vectobac^®^ (Shandong Lukang Shelile Pharmaceutical Co. Ltd China) water granules. Using *Bti* with *Ae. aegypti* from West Kalimantan at a concentration of 0.02 caused 89% larval mortality within 9 h of exposure [69]. *Bacillus thuringiensis* is relatively safe for nontarget animals, particularly when used in small doses, and is environmentally friendly [61, 65, 70]. The use of bioinsecticides is recommended to mitigate the effects of chemicals. Long-term exposure to *Bti* did not induce resistance, nor was there cross-resistance with temefos [66]. *Bacillus thuringiensis* represents a viable bioinsecticide alternative for controlling *Ae. aegypti* and *Ae. albopictus* larvae [67].

### Wolbachia-induced cytoplasmic incompatibility

*Wolbachia pipientis*, a bacterium residing in the guts of most terrestrial arthropods, has shown potential for inhibiting the transmission of vector-borne diseases. The Sindbis virus is endemic in Sweden, where it is predominantly transmitted by *Cx. torrentium* *C. trentium*, followed by *Cx. quinquefasciatus*.

*Wolbachia* is maternally inherited bacteria and can alter host reproduction, leading to cytoplasmic incompatibility (CI). Cytoplasmic incompatibility refers to sperm–egg incompatibility that results in embryonic mortality. *Wolbachia* are being explored for vector control due to their sterilizing effect on mosquitoes, particularly in the *Cx. quinquefasciatus* complex, which are important vectors for arboviruses, filarial nematodes, and avian malaria [71]. *Wolbachia* infections in *Ae. albopictus* provide an interesting model for studying CI and population replacement [72].

Wolbachia wAlbB showed a strong cytoplasmic incompatibility (CI) effect, as evidenced by the absence of egg hatching in crosses between Wolbachia-infected male Aedes aegypti and wild-type (uninfected) female Aedes aegypti in laboratory and fieldwork experiments. Wolbachia infection had no significant impact on general fitness, fertility, body size (female and male) and mating competitiveness of new hosts [73]. CI is the most common form of *Wolbachia*-induced reproductive alteration in insects [74]. *Wolbachia* endosymbionts are successful insect colonizers. Some strains of *Wolbachia* induce CI in host insects, causing *Wolbachia*-infected males to produce inviable offspring when mating with uninfected females [75].

Initially described in *Culex. quiquefasciatus* mosquitoes, CI manifests as a conditional embryonic lethality when males infected with CI-inducing *Wolbachia* strains mate with uninfected females (unidirectional CI) or with females carrying other incompatible *Wolbachia* strains [75].

We observed an unusual pattern of CI observed in crossing experiments between the ARwP and naturally infected males (SR/superinfected Rome lines). AR*w*P is a wPip (strain of *Wolbachia* which was identified from the *Cx. pipiens* mosquito species) *Wolbachia*-infected *Ae. albopictus* and exhibits bidirectional incompatibility with wild types. Specifically, AR*w*P males induce full sterility in wild-type females throughout most of their lifetimes. In contrast, crosses between SR males and AR*w*P females become partially fertile with male aging. We showed that the observed decrease in CI penetrance with increasing age in SR males is associated with a decline in *Wolbachia* density, particularly the *w*AlbA strain that occurs in older, coinfected males [74].

This is the first observation of *Wolbachia*-induced amplification of human pathogens in mosquitoes, emphasizing the need for precautions before releasing *Wolbachia*-infected insects as part of control programs for vector-borne diseases [75]. *Culex quiquefasciatus* is among the most important mosquito species because females can carry pathogens, which have had a significant impact worldwide, making this species an important target for control efforts [76].

*Wolbachia* efficacy trials in Indonesia commenced in Jogjakarta and parts of the Bantul region in 2017. These trials were conducted in experimental areas with a population of around 312,000 people over 27 months, involving 8144 participants aged 3–45 years [77]. This approach resulted in a 77% reduction in dengue cases in Yogyakarta and an 86% decrease in hospitalized patients [78]. The successful implementation in Yogyakarta serves as a model for other regions in Indonesia, with considerations for equipment, human resources, budget availability, and regulatory aspects, all aimed at reducing cases of dengue fever transmitted by *Ae. aegpti* and *Ae. albopictus* mosquitoes.

### Insect growth regulators as chemical larvicides

Insecticides, which are chemical compounds widely used to control insect populations, encompass larvicides that specifically target insect larvae. In recent years, insect growth regulators (IGRs) have gained popularity as safe and effective alternatives to traditional insecticides. Insect growth regulators disrupt insects’ normal growth and development, preventing them from reaching adulthood and reproducing. This review examines the use of IGRs as chemical larvicides, focusing on their direct application and autodissemination.

Studies have shown that pyriproxyfen IGR is highly effective against *Ae. albopictus* larvae, with a mortality rate of 99.6% after 24 h of exposure [79]. Notable changes, such as cuticle peeling to the chest and swelling of the head and chest, were observed after exposure [80]. Similarly, methoprene IGR was effective against *Anopheles stephensi* larvae [81], and diflubenzuron IGR was effective against *Cx. quinquefasciatus* larvae. These findings suggest that IGRs can prevent the development of larvae into adult mosquitoes [82]. One advantage of direct application is that it allows for precise targeting of breeding sites. Overall, the direct application of IGRs exhibits promising potential as a safe and effective method for controlling mosquito populations, particularly in areas where other control methods may be impractical or unsafe.

Autodissemination involves using adult mosquitoes to spread IGRs to breeding sites. In this method, adult mosquitoes are lured to an IGR-containing attractant, subsequently transferring it to breeding sites when they lay their eggs. Automated deployment has several advantages over live applications, including cost reduction and increased coverage [83]. Several studies have investigated the efficacy of autodissemination with IGRs; Pyriproxyfen autodissemination, for instance, exhibited significant efficacy against *Ae. albopictus*, leading to an 89.9% reduction in the larvae count [84]. Another study investigated pyriproxyfen IGR autodissemination for *Ae. aegypti* control, noting significant reductions in adult mosquito populations and breeding sites [85].

One potential limitation of autodissemination is that it requires adult mosquitoes to transport IGRs to breeding sites. In addition, the effectiveness of autodissemination is influenced by environmental factors, such as rainfall or temperature, which can affect the movement and behavior of adult mosquitoes [86]. Despite these limitations, IGR autodissemination holds considerable promise as a safe and effective method for controlling insect populations, particularly in urban and suburban areas where traditional control methods might be less effective.

## Evaluation of Control Methods in Large-Scale Field Trials

### Monitoring of *Aedes* mosquitoes

The monitoring of *Aedes* mosquitoes plays a crucial role in controlling and preventing mosquito-borne diseases, such as dengue, Zika, and chikungunya viruses. As these diseases are transmitted by *Aedes* mosquitoes, monitoring their populations can help identify high-risk areas and guide control efforts. Monitoring methods for *Aedes* mosquitoes include trapping, oviposition traps, and larval surveys.

Trapping is a common method for monitoring adult *Aedes* mosquitoes. These traps use attractants such as CO_2_, octenol, and other chemicals to lure and capture mosquitoes. The different types of traps available include sticky, gravid, and Centers for Disease Control light traps. Studies have shown that trap data can be used to estimate mosquito abundance and predict disease transmission [87]. Oviposition traps represent another monitoring approach. These traps use water and a substrate for mosquitoes to lay their eggs on, which are subsequently collected and counted. Oviposition traps help monitor the presence and abundance of *Aedes* mosquitoes and identify breeding sites [88]. Finally, larval surveys present an alternate method for monitoring immature *Aedes* mosquitoes. These surveys involve inspecting potential breeding sites, such as water-filled containers, and recording larvae presence and abundance. Larval surveys can help identify areas at risk of *Aedes* mosquito infestation and guide control efforts [89]. In addition to these methods, new technologies, such as remote sensing and geographic information systems, are being developed and tested for *Aedes* mosquito monitoring [89].

Overall, monitoring *Aedes* mosquitoes is essential for controlling and preventing mosquito-borne diseases. Different monitoring methods can be used, depending on specific goals and available resources. In addition, consistent surveillance and monitoring can provide critical information for guiding control efforts.

### Implementing an integrated control strategy for IMS

Implementing an integrated IMS control strategy in Indonesia requires careful consideration of the target species, ecology, public health, and disease transmission dynamics [1]. To prevent disease transmission, using insecticides and eliminating small breeding sites near infected areas is recommended, as demonstrated in Indonesia to prevent chikungunya or dengue virus transmission by *Ae. albopictus* [25]. On the other hand, timing and treatment are critical if an integrated IMS control strategy aims to achieve medium- to long-term population reduction to reduce the risk of bite injury and arbovirus outbreaks due to population fluctuations in the target species. For example, methods such as insecticide spraying are more effective when mosquito populations are dense or cover vast areas [35]. Efficacy, specificity, residual effect, resistance selectivity, and ecological impact should be considered when choosing a control method. For example, the use of larvicides is among the most effective methods when treatment is focused on key breeding sites in an area. However, this may vary among urban, suburban, and rural areas [90]. Time-consuming and resource-intensive reduction methods should involve communities through a community-based approach. Overall, the success of an integrated IMS control strategy depends on cooperation among policymakers, authorities, academia, and the public. Furthermore, the implementation of such a strategy should be adapted to available financial and human resources [23].

## Conclusion

Implementation and evaluation of an integrated STI control strategy against *Aedes* mosquitoes has been carried out in Indonesia. Some of the STI surveillance methods discussed in this observation have been used successfully, but are limited to local locations. Approaches such as the *Wolbachia* technique showed promising results in Yogyakarta and were later developed in five other regions, so this technology supports potential future use on a larger scale.

Evaluation of *Aedes* density requires entomological surveys to monitor STIs. This allows the effectiveness of control methods to be evaluated by determining whether adult populations and/or egg numbers are reduced at treatment sites compared to control sites. Finally, mapping and modeling should be developed to optimize integrated STI control strategies, and cost-effectiveness analyses should be conducted to guide policy. In conclusion, there are various methods of vector control against the *Aedes* mosquito. Traditional methods such as source reduction, community education and routine use of insecticides are implemented by the government to reduce *Aedes* populations but have limited success, perhaps due to low community participation, and lack of coordination and synchronization of implementation. The involvement of the government, community, and academics needs to be carried out to accelerate comprehensive, effective, and efficient control.

## Authors’ Contributions

MRR, LM, and TZ: Conceptualization, writing original draft, and visualization. JW, NAF, DR, NR, MN, ID and AS: Analysis and interpretation of data and drafted and revised the manuscript. TZ and LM: Critically reviewed and edited the manuscript. All authors have read, reviewed, and approved the final manuscript.
